# THE ASSOCIATION BETWEEN LONELINESS AND CHILDLESSNESS IN MIDDLE TO LATE LIFE: DOES FRIENDSHIP AND GENDER PLAY A ROLE?

**DOI:** 10.1093/geroni/igae098.1964

**Published:** 2024-12-31

**Authors:** Alison Rataj, Andrew Alberth, Yan-Jhu Su, Elisabeth Stam, Jeffrey Stokes

**Affiliations:** University of Massachusetts Boston, Durham, New Hampshire, United States; University of Massachusetts Boston, Boston, Massachusetts, United States; University of Massachusetts Boston, Boston, Massachusetts, United States; University of Massachusetts Boston, Boston, Massachusetts, United States; University of Massachusetts Boston, Boston, Massachusetts, United States

## Abstract

**Objective:**

Loneliness is a risk factor for negative health outcomes; however, little attention has been paid to the role of parental status as a contributor to loneliness in later life. Limited research has explored how this association may vary based on levels of friend support, friend strain, and gender. This study addresses these gaps by exploring whether those who are childless, have less friend support or friend strain, and are female are at a higher risk of loneliness.

**Methods:**

This cross-sectional study used pooled data from 11,900 older adults 50 years and older from the 2016 and 2018 waves of the Health and Retirement Study. Loneliness was assessed across parental status groups, friendship support and friendship strain scales, and gender using ordinary least squares regression. Multiple imputation was conducted to address missing data.

**Results:**

Those who did not have children (n=1,444, 13.0%) were substantially lonelier than those with children in adjusted models. Friend support was associated with reduced loneliness, particularly for childless adults when compared to parents. Midlife and older men were lonelier than women overall, and interaction effects show that childlessness played a larger role in shaping loneliness among women than men. Loneliness did not differ across levels of friendship strain.

**Discussion:**

Parental status and gender are intertwined concerning their effects on loneliness. With the changing demographics of “family”, future research should identify specific categories of parental status (i.e., did not want children, blended families) and how the mechanics of friend support (i.e., how much, what types) impact loneliness.

